# Regulatory network of metformin on adipogenesis determined by combining high-throughput sequencing and GEO database

**DOI:** 10.1080/21623945.2021.2013417

**Published:** 2022-01-03

**Authors:** Zhicong Zhao, Chenxi Wang, Jue Jia, Zhaoxiang Wang, Lian Li, Xia Deng, Zhensheng Cai, Ling Yang, Dong Wang, Suxian Ma, Li Zhao, Zhigang Tu, Guoyue Yuan

**Affiliations:** a Department of Endocrinology, Affiliated Hospital of Jiangsu University, Zhenjiang, Jiangsu, China; b Department of Endocrinology, Suzhou Municipal Hospital, Suzhou, Jiangsu, China; c School of Life Sciences, Jiangsu University, Zhenjiang, Jiangsu, China

**Keywords:** Adipogenensis, metformin, transcriptome, gene expression omnibus, integrated bioinformatics

## Abstract

Adipose differentiation and excessive lipid accumulation are the important characteristics of obesity. Metformin, as a classic hypoglycaemic drug, has been proved to reduce body weight in type 2 diabetes, the specific mechanism has not been completely clear. A few studies have explored its effect on adipogenesis in vitro, but the existing experimental results are ambiguous. 3T3-L1 preadipocytes were used to explore the effects of metformin on the morphological and physiological changes of lipid droplets during adipogenesis. A high throughput sequencing was used to examine the effects of metformin on the transcriptome of adipogenesis. Considering the inevitable errors among independent experiments, we performed integrated bioinformatics analysis to identify important genes involved in adipogenesis and reveal potential molecular mechanisms. During the process of adipogenesis, metformin visibly relieved the morphological and functional changes. In addition, metformin reverses the expression pattern of genes related to adipogenesis at the transcriptome level. Combining with integrated bioinformatics analyses to further identify the potential targeted genes regulated by metformin during adipogenesis. The present study identified novel changes in the transcriptome of metformin in the process of adipogenesis that might shed light on the underlying mechanism by which metformin impedes the progression of obesity.

## 1. Introduction

Lipid is an important component of cells and tissues in the body, such as various plasma membranes and nerve myelin sheath, mainly obtained through exogenous food and biosynthesis in vivo [1]. Adipose tissue is a dominant place for lipid storage among various tissues, an important regulator of the body’s energy balance. It contains a variety of cell types with fat cells as the main body. The current studies considered adipogenesis may lead to over-modification of adipose tissue, changing the morphology and function of individual adipocytes and the entire adipose tissue. It causes alteration in the size and number of adipocytes, inflammation of adipose tissue, pathological remodelling of extracellular matrix and lipid synthesis, thus leading to fibrosis and the secretion of adipokines [1–4]. Under pathological conditions, abnormal adipogenesis may lead to hyperlipidaemia, atherosclerosis, non-alcoholic fatty liver, tumours and neurodegenerative diseases [5,6]. Therefore, an in-depth study of the molecular mechanisms controlling the adipogenesis will help us better understand the pathogenesis and pathophysiology of metabolic syndrome, diabetes and cardiovascular diseases.

Metformin has been used to reduce fasting blood glucose in type 2 diabetes for the improvement of insulin sensitivity [7–9]. Meanwhile, it also attracts wide attention due to its multifunctional effects, including reducing appetite, preventing cardiovascular disease, improving endothelial function, regulating inflammatory response, preventing cancer, regulating glucose and lipid metabolism and reducing body weight [10]. The potential function of metformin in mouse 3T3-L1 cells has been evaluated, and some researchers found that metformin could inhibit adipogenesis to cause the pattern of lipid synthesis [11–16]. Several studies showed that metformin might affect adipocyte differentiation by acting on the key enzymes of lipid synthesis [17–19]. In addition, some studies also indicated that metformin, alone or in combination with other molecules, could finely modulate the inflammatory response and autophagy in the adipose tissue [20–23]. It is widely recognized that AMPK signal is indispensable in metformin regulating adipogenesis [16,24–26]. However, the deeper mechanism of metformin inhibiting adipocyte differentiation has not yet been clarified. In recent years, bioinformatics has been widely applied in the research of various diseases, especially the use of Gene Expression Omnibus (GEO) to screen potential target genes. Making full use of these big data sets provided a bright value for life science research. In view of the inevitable mistakes in independent experiments, researchers integrated the results of various experiments to accurately identify differential genes and clarify the main molecular mechanisms [27–29]. Therefore, transcriptome profile combined with GEO data can further explore the detailed mechanism of metformin regulating adipogenesis.

In the current study, we firstly profiled the transcriptome changes occurred in response to adipogenesis with metformin treatment by using high-throughput sequencing. Meanwhile, we reanalysed and integrated the results of seven expression profile data sets from a public expression database to reveal the differentially expressed genes (DEGs) associated with adipogenesis regulated by metformin more accurately. With these differential expression genes, then some bioinformatics analysis and biological verification were carried out. The data provided new insights for a comprehensive understanding the potential mechanism of metformin acting on the process of adipogenesis.

## 2. Results

### 2.1 Effects of metformin on adipogenesis in 3T3-L1 preadipocytes

**Figure 1.**
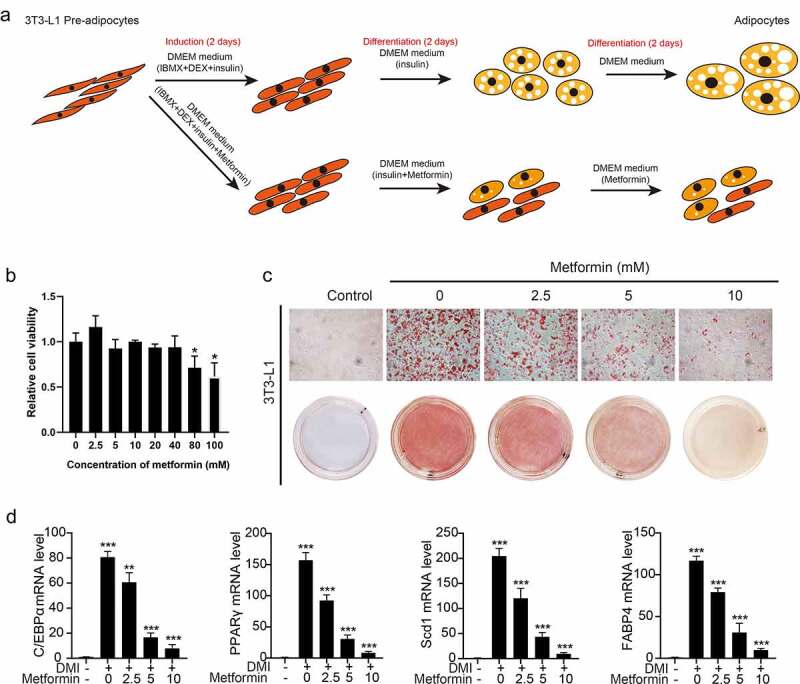
Metformin inhibits adipogenesis in 3T3-L1 preadipocytes. (a) the schematic diagram of metformin intervention in 3T3-L1 adipocyte differentiation was shown. (b) 3T3-L1 cells were exposed to 0–100 mM of metformin for 48 h, and cell viability evaluated by MTT assay were shown. (c) Graphs of differentiated 3T3-L1 adipocytes stained by Oil Red O for various concentration of metformin treatment. Scale bar indicates 100 μm. (d) Quantitative PCR analysis of mRNA of the adipogenic genes PPARγ, C/EBPα, Scd1 and FABP4 in the process of metformin intervening adipogenesis. Statistical significance was calculated by using two-tailed Student’s t test for the effect of metformin on adipogenesis. *P < 0.05; **P < 0.01; *** *P* < 0.001.

Firstly, the schematic diagram of metformin intervention in 3T3-L1 adipocyte differentiation was shown (Figure 1a). We explored the effects of metformin on adipocyte differentiation at different concentration. As shown in Figure 1c, the process of adipogenesis was inhibited with 2.5, 5 and 10 mM of metformin. 2.5–40 mmol/L concentrations of metformin did not inhibit the growth of 3T3-L1 cells (Figure 1b). In the DMI group, characteristic morphological changes and obvious increase of lipid droplets occurred during 3T3-L1 adipocyte differentiation, indicating that differentiation model was carried out successfully. Metformin visibly relieved the morphological and functional changes. Considering that the process of adipogenesis involves a network of genes and is regulated by a cascade of transcription factors, we examined expressions of related transcriptional factors during the adipogenesis upon treating with/without various concentration of metformin. Consistent with the morphological and functional changes, our data showed that marker genes related to adipose differentiation (C/EBPα, PPARγ, Scd1 and FABP4) were inhibited by various concentration of metformin (Figure 1d), which further confirmed the inhibition of metformin on 3T3-L1 adipocyte differentiation. To explore the mechanism of metformin on adipocyte differentiation, preadipocytes, vehicle treated differentiated adipocytes, and differentiated adipocytes treated 5 mM metformin were collected to conduct RNA-seq.

### 2.2 Characterization of the gene expression profile of 3T3-L1 preadipocytes under metformin treatment

**Figure 2.**
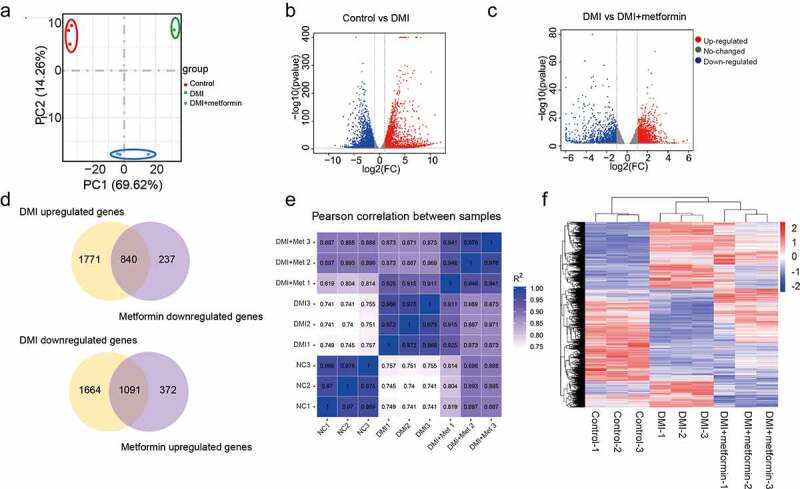
A number of differentially expressed genes in metformin inhibiting 3T3-L1 preadipocytes differentiation. (a) Principal Component Analysis (PCA) of three independent samples for control, DMI and DMI+ metformin groups. (b-c) Volcano plot for the Control vs DMI (b) and DMI vs DMI+ metformin. (d) A Venn diagram presents the number of DEGs that mediated metformin inhibiting adipognensis is unique or shared between adipogenesis-responsive genes and metformin-responsive genes. (e) Pearson correlation is shown between samples. (f) Heatmap of the DEGs in the three groups. Each row represents one gene, and each column represents one sample. Red indicates that the expression of genes is relatively up-regulated, and green indicates that the expression of genes is relatively down-regulated.

Transcriptome studies on 3T3-L1 preadipocytes intervened with 5 mM metformin during the process of adipogenesis were performed using GeneChip Human Gene 1.0 ST arrays. Here, principal component analysis (PCA) was applied to the transcriptome profiles of these coding genes to test whether different sample groups could be clearly separated by their transcriptome characteristics. As shown in Figure 2a, the first two principal components (PC1 and PC2) can clearly separate the three groups of samples, which explains the changes of 69.62% and 14.28%, respectively, suggesting the significant difference among the 3 groups of samples. The purpose of differential expression gene screening is to find out the DEG between groups and further analyse its function. In parallel with the changes of adipogenic marker genes and morphology, the transcriptome results showed that there were 2609 genes up-regulated and 2752 genes down-regulated in the adipogenesis process of 3T3-L1 cells (Figure 2b). There were 1462 genes up-regulated and 1077 genes down-regulated in the process of metformin intervening adipogenesis (Figure 2c). among the differential expressed genes, 840 genes were upregulated by DMI and downregulated by metformin, 1091 genes were downregulated by DMI, and upregulated by metformin(Figure 2d). Moreover, we also calculated the correlation value between each sample based on the normalized expression results and drew a correlation heatmap (Figure 2e-2f), suggesting metformin reversed the expression pattern of genes related to differentiation at the transcriptome level.

### 2.3 Gene Ontology analysis of the differential expression genes

**Figure 3.**
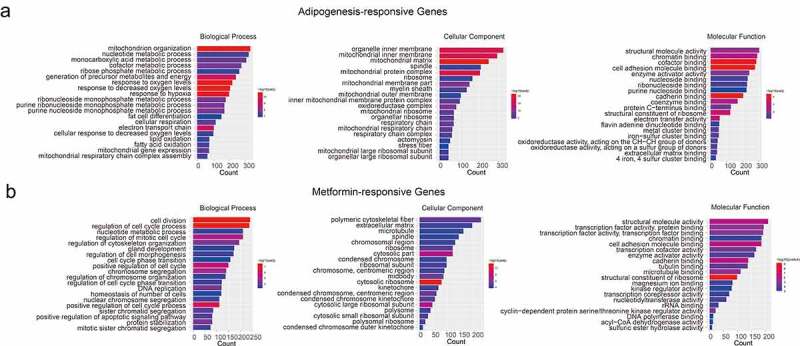
Gene ontology analysis of the adipogenesis-responsive and metformin-responsive genes. (a) GO enrichment analysis of adipogenesis-responsive genes. It showed the top 20 significantly enriched GO terms including biological process, cellular component, and molecular function. (b) The GO enrichment analysis of metformin-responsive genes as above indicative operation.

In order to better understand the related function of DEGs in metformin intervention of 3T3-L1 adipogenesis, Gene Ontology (GO) analysis was used for enrichment analysis and classification according to the biological process, cellular component, and molecular function. GO analysis of ‘Adipogenesis-responsive genes’ identified enriched functions was defined associated with ‘cofactor metabolic process,’ ‘electron transport chain,’ ‘generation of precursor metabolites and energy,’ ‘mitochondrion organization,’ ‘cadherin binding,’ and ‘cell adhesion molecule binding,’ implying that the cofactor metabolic process and intracellular signalling transduction are the major molecular functions during 3T3-L1 adipogenesis (Figure 3a). Metformin-responsive genes were involved in ‘cell division,’ ‘regulation of cell cycle,’ ‘condensed chromosome kinetochore,’ ‘cadherin binding,’ and ‘cell adhesion molecule binding,’ that demonstrated above processes might be in involved in metformin inhibiting adipogenesis (Figure 3b).

### 2.4 Analysis of important KEGG pathways

**Figure 4.**
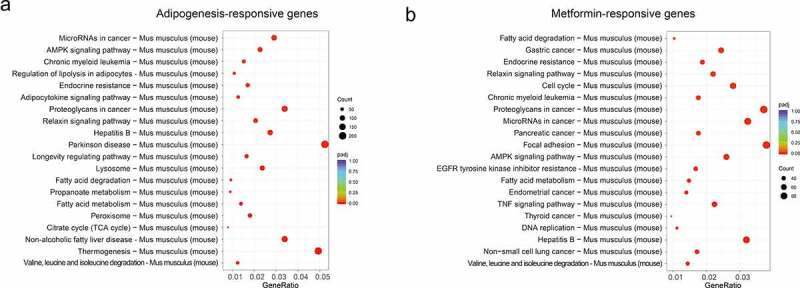
KEGG analysis of the adipogenesis-responsive and metformin-responsive genes. (a) KEGG analysis of the adipogenesis-responsive genes. It showed the top 20 significantly enriched KEGG pathways. The rich factor as the abscissa and the KEGG terms as the ordinate are plotted. (b) KEGG analysis of the metformin-responsive genes as above indicative operation.

We next used the differential genes for the KEGG pathway enrichment using clusterProfiler R package (http://www.genome.in/kegg/) [30]. The differential genes were significantly enriched in the classifications of ‘MicroRNAs in cancer,’ ‘AMPK signaling pathway,’ ‘Fatty acid metabolism,’ ‘Fatty acid degradation,’ ‘Endocrine resistance’, ‘Relaxin signaling pathway’, ‘Hepatitis B’, ‘Valine, leucine and isoleucine degradation’, and ‘Chronic myeloid leukemia’ in the whole process of adipocyte differentiation (Figure 4a). In addition to the ‘MicroRNAs in cancer,’ ‘AMPK signaling pathway,’ ‘Fatty acid metabolism,’ ‘Fatty acid degradation,’ ‘Endocrine resistance’, ‘Relaxin signaling pathway’, ‘Hepatitis B’, ‘Valine, leucine and isoleucine degradation’, and ‘Chronic myeloid leukemia’ were also enriched in the metformin inhibiting adipocyte differentiation (Figure 4b). These results suggested that the above processes might be involved in the metformin inhibiting adipocytes differentiation.

### 2.5 Identification of DEGs associated with metformin inhibiting adipogenesis using integrated bioinformatics

**Figure 5.**
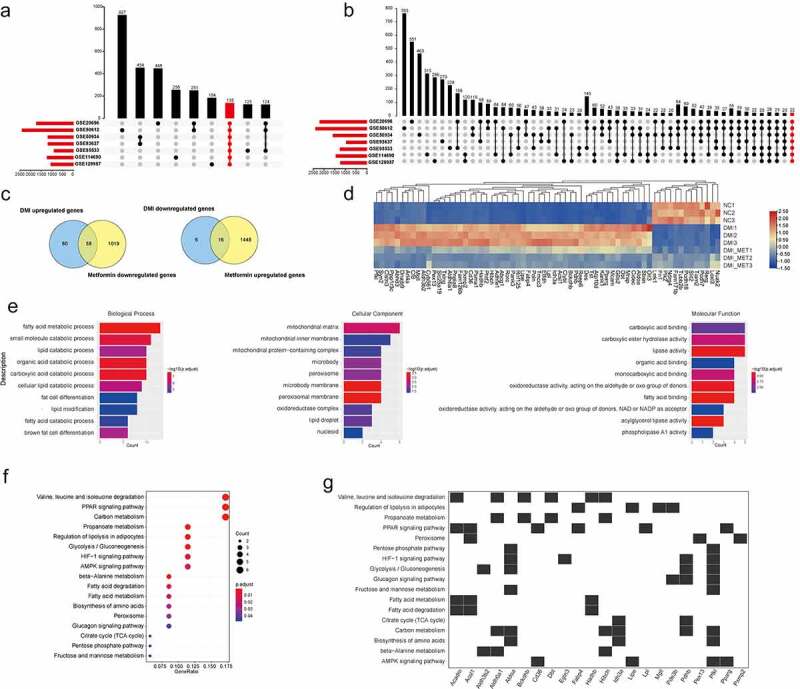
Identification of candidate genes associated with metformin inhibiting adipogenesis. (a-b) the screening and identification of upregulated genes (a) and downregulated genes (b) related to adipose differentiation by integrating the seven adipogenesis data set of GEO database. (c) A Venn diagram presented the number of DEGs that mediated metformin inhibiting adipogenesis was unique or shared (d) Heat maps show the 74 differentially expressed genes up-regulated or down-regulated by DMI and reversed by metformin after integrated analysis. (e) GO enrichment analysis of integrated DEGs. It showed the top 10 significantly enriched GO terms including biological process, cellular component, and molecular function. (f-g) KEGG analysis of integrated DEGs is shown.

To further identify DEGs associated with metformin inhibiting adipogenesis more accurately, we selected seven expression profile datasets relevant to adipogenesis from a public expression database (Table 1), and then integrated these results. Through rank analysis, we identified 138 common upregulated genes and 22 downregulated genes relevant to adipogenesis among the raw datasets (Figure 5a, 5b). For considering that metformin might inhibit 3T3-L1 adipogenesis by regulating the expression of unclear target gene. Hence, we further integrated and analysed the transcriptional data of metformin intervention in adipogenesis, among which 74 genes might be identified to mediate metformin suppressing adipocyte differentiation (Figure 5c,5d). For the further Gene Ontology (GO) analysis of candidate genes, our results demonstrated that the integrated genes were mainly involved in ‘fatty acid metabolic process’ and ‘multifarious catabolic process’ (Figure 5e). In addition, KEGG analysis showed that they were mainly associated with the ‘valine, leucine and isoleucine degradation’ ‘PPAR signaling pathway’ and ‘carbon metabolism’(Figure 5f,5g).

**Table 1.** Undifferentiated and differentiated 3T3-L1 cell samples from the NCBI Gene Expression Omnibus (GEO) database used in this study

Reference	CEO data	Platform	Differentiation Stage of Sample		Number of DEGs (up/down)
			Undifferentiated	Differentiated	
Mikkelsen, T.S.et al. (2010) [36]	GSE20696	GPL1261	GSM519581 GSM519582	GSM519585 GSM519586	3949 (1744/2205)
Romero, M. et al. (2018) [37]	GSE93637	GPL1261	GSM2459304 GSM2459305 GSM2459306	GSM2459310 GSM2459311 GSM2459312 GSM2459313	1942 (971/971)
Duteil, D. et al. (2014) [38]	GSE50934	GPL13112	GSM1232686 GSM1232687 GSM1232688	GSM1232689 GSM1232690 GSM1232691	2795 (1206/1589)
Siersbæk, R. et al. (2017) [39]	GSE95533	GPL18480	GSM2515916 GSM2515917	GSM2515922 GSM2515923	2186 (922/1264)
Al, A.H. et al. (2015) [40]	GSE50612	GPL13112	GSM1224678 GSM1224679	GSM1224682 GSM1224683	4811 (2410/2401)
Kou Y.et al. (2019) [41]	GSE114690	GPL21273	GSM3147313 GSM3147314 GSM3147315	GSM3147319 GSM3147320 GSM3147321	2695 (1240/1455)
Wuping, S.et al. (2020) [42]	GSE129957	GPL13112	GSM3728574 GSM3728575 GSM3728576	GSM3728580 GSM3728581 GSM3728582	2312 (1067/1245)

### 2.6 Protein–Protein Interaction (PPI) network construction and tissue expression profile analysis of DEGs interacting with adipogenesis marker genes

**Figure 6.**
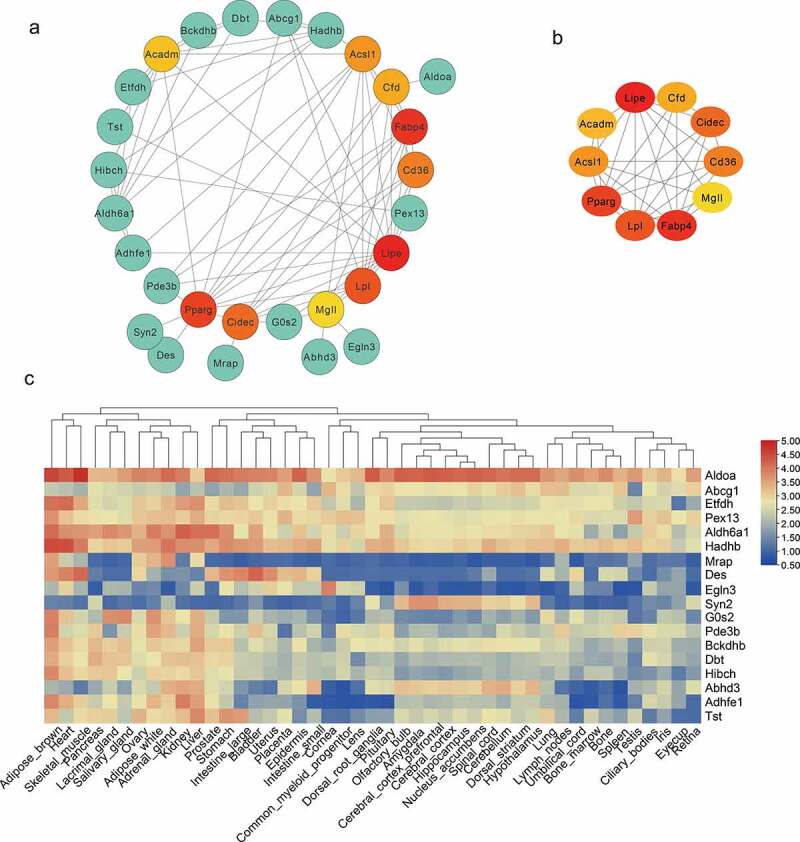
Protein–Protein Interaction (PPI) and tissue expression profile analysis of integrated DEGs. (a) Using Cytoscape software, the PPI network was visualized (isolated nodes were removed). (b) Top 10 genes with the highest degrees in the PPI network. (c) Tissue expression heat maps of these candidate genes is demonstrated through the BioGps database.

To analyse the interaction among DEG expression products, the Search Tool for the Retrieval of Interacting Genes/Proteins (STRING) database was used to construct a PPI network. A total of 71 nodes and 103 edges were obtained with a combined score >0.4, as shown in Figure 6a (isolated nodes were ignored). The top 10 highest degree nodes were shown in Figure 6b. Among these genes, the integrated genes contained many fat marker genes (such as LPL, peroxisome proliferator activated receptor gamma (PPARγ), FABP4). Considering that candidate genes interacting with fat marker genes are more likely to be involved in fat formation, we analysed the tissue expression profile of these genes through the BioGps database (http://biogps.org/#goto=welcome). As shown in Figure 6c, some genes are expressed in specific tissues, e.g, Marp and Hibch are highly expressed in brown adipose and liver tissue, G0s2 is widely expressed in adipose tissues.

### 2.7 Validation and differential expression analysis of integrated DEGs in obese mice

**Figure 7.**
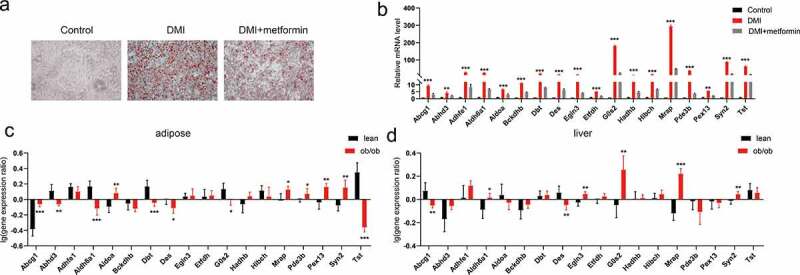
Validation and differential expression analysis of integrated DEGs in obese mice. (a) Graphs of differentiated 3T3-L1 adipocytes stained by Oil Red O for 5 mM metformin treatment. (b) The mRNA levels of integrated DEGs were assessed by real-time PCR as shown in A. (c-d) The expression difference of these genes were analysed in adipose tissue (c) and liver (d) of lean and ob/ob mice from the attie lab diabetes database. Statistical significance was calculated by using two-tailed Student’s t test for the effect of metformin on adipogenesis. *P < 0.05; **P < 0.01; *** *P* < 0.001.

The role of integrated DEGs interacting with adipogenesis marker genes is still unclear. Therefore, we validated the mRNA expression levels of these genes in the process of metformin inhibiting adipogenesis using quantitative RT-PCR. Our results demonstrated that the mRNA expression levels of these genes were significantly increased in the process of adipogenesis. On the other hand, metformin decreased the expression of these genes (Figure 7b). Considering the correlation between fat differentiation and obesity, we further analysed the mRNA expression of these genes in the adipose tissue and liver of ob/ob mice from the attie lab diabetes database (http://diabetes.wisc.edu/search.php) [31]. As shown in Figure 7c,7d. Abcg1, Aldoa, Mrap, Pex13 and Syn2 are significantly increased, Abhd3, Aldh6a1, Dbt, Des, G0s2 and Tst are significantly decreased in adipose tissue of ob/ob mice compared with lean mice. In liver, Aldh6a1, Egln3, G0s2, Mrap and Syn2 are significantly increased, whereas Abcg1 and Des are dramatically decreased, compared with lean mice. These results suggested that those of Abcg1, Aldoa, Mrap, Pex13, Syn2, Aldh6a1, Egln3 and G0s2, especially Mrap and Syn2, might be involved in the regulation of adipocyte differentiation and obesity, and regulated by metformin.

## 3. Discussion

Moderate amount of lipid is very important for human homoeostasis. There is too little lipid, it may cause osteoporosis, hair loss, memory decline, abnormal metabolism of growth hormone, endocrine disorder and amenorrhoea. The excessive accumulation of lipids can directly lead to obesity and induce non-alcoholic fatty liver disease, type 2 diabetes, cardiovascular disease, cancer and other diseases [32,33]. Among these diseases, obesity is one of the major health crises in the world. In addition, various diseases induced by obesity have become an important reason affecting people’s health and quality of life [34]. Obesity is characterized by the large amounts of lipid accumulation and increase of adipose tissue mass, which is caused by the increase of adipocyte size (hypertrophy) and number (proliferation), indicating that adipocytes play a major role in obesity [35]. The present studies generally considered that adipogenesis is one of the significant processes of increasing the number of adipocytes. Therefore, in-depth analysis of the molecular mechanism of fat differentiation is expected to find the molecular target of obesity

In the past decades, metformin had been reported to exert beneficial effects on various diseases beyond diabetes. Such as reducing appetite and body weight, preventing cardiovascular disease, improving endothelial function, regulating inflammatory response, preventing cancer, regulating glucose and lipid metabolism [10]. More recently, the role of metformin in mouse 3T3-L1 cell line was evaluated, and some researchers have found that metformin inhibited adipogenesis. Several studies have shown that metformin might regulate adipogensis by acting on the key enzymes of lipid synthesis [11–16]. In addition, some studies also indicated that metformin, alone or in combination with other molecules, could finely modulate the inflammatory response and autophagy in the adipose tissue [20–23]. It is generally recognized that metformin plays biological function through activating AMPK signal pathway [16,24–26], and there are few studies on the deeper molecular mechanism of metformin inhibiting adipocytes differentiation. It is of great significance and necessity to evaluate the impact of metformin on adipogenesis. Hence, we depicted the impact of treatment with metformin on the transcriptomic in preadipocytes differentiation.

In the present study, we characterized the effect of metformin on the transcriptomic response to the process of adipogenesis. Our results indicated that metformin induced substantial transcriptional changes of differentiation differential expression genes related to adipogenesis involved in the multiple biological signal. Previous studies identified gene expression profiles during adipogenesis and identified a large number of differentially expressed genes related to the initiation of adipogenesis. Considering the existence of experimental differences for each independent sample, we selected seven expression profile datasets relevant to adipogenesis from a public expression database and then integrated these results [36–42]. Through rank analysis, a total of 74 DEGs related to adipogenesis regulated by metformin were identified. The candidate genes list contained many fat marker genes (such as LPL, peroxisome proliferator activated receptor gamma (PPARγ), FABP4), and a large number of adipogenic differentiation studies have been carried out on them, many of the other genes might be involved in the regulation of adipocyte differentiation and obesity.

It is known that metformin has potent lipid and glucose-lowering function, mainly by activating the AMPK signalling pathway in diabetic subjects [16,25,26]. Given that the possibility of metformin acting on other signalling pathways to exert beneficiary effect on lipid metabolism, the functional annotation of DEGs was performed to fully understand the processes and pathways in which they participate. GO analysis revealed that fatty acid metabolic and catabolic process were involved in metformin inhibiting adipogenesis. KEGG analysis of DEGs showed other signalling pathway in which they were involved, beside AMPK signalling pathway, such as ‘Valine, leucine and isoleucine degradation’, ‘HIF-1 signaling pathway’ and ‘Glucagon signaling pathway’, suggesting that metformin regulated multiple signalling pathway to inhibit the process of adipogenesis.

A PPI network of integrated DEG-encoded proteins is constructed and the 10 closely adipogenesis and lipid metabolism-related genes are well-known as key genes in fat differentiation and lipid metabolism functions, such as the transcriptional regulation of adipogenesis (Pparg), fatty acid transport (fatty acid binding protein 4 (Fabp4), CD36 Molecule (Cd36), acyl-CoA synthetase long-chain family member 1 (Acsl1), lipoprotein lipase (Lpl), lipase, hormone sensitive (Lipe) [43,44]. In addition, 18 candidate genes are closely interacting with adipogensis marker genes, many of these play an unknown role in the process of metformin inhibiting adipogenesis. Further expression patterns of these candidate genes were analysed in liver and adipose tissue of obese mice through the attie lab diabetes database [31]. Abcg1, Aldoa, Mrap, Pex13, Aldh6a1, Egln3, G0s2 and Syn2 are significantly increased in adipose or liver tissue of ob/ob mice compared with lean mice, suggesting these genes are associated with obesity, are very rarely reported to be related to adipogenesis. Adipocyte hypertrophy occurs in obesity, it is possible that this cellular distention upregulated these genes and further leads to the changes in gene programs. Therefore, the roles of these screened genes in the regulation of adipocyte differentiation and obesity might involve a cellular distention mechanism.

Meanwhile, we verified the expression changes in some key genes, metformin inhibited adipogenesis with downregulating key genes associated with adipogenesis and obesity, suggesting metformin might play potent lipid and glucose-lowering function by targeting these genes. Further studies are warranted to clarify this. This study has greatly narrowed the range of metformin regulated potential key genes and provides high-value targets for metformin on subsequent adipogenic differentiation research.

## 4. Materials and methods

### 4.1 Cell culture and treatment

Mouse embryonic fibroblast 3T3-L1 cells were purchased from the American Type Culture Collection (Manassas, VA) and cultivated in DMEM (Gibco, Gaithersburg, MD, USA) containing 10% FBS and 1% penicillin/streptomycin (Life Technologies, Grand Island, NY, USA). 3T3-L1 preadipocytes were seeded into six well plates when contact inhibition occurred. Then, cells were induced by 1-methyl-3-isobutyl xanthine, dexamethasone and insulin according to Anil Kumar’s method [45]. To examine the effect metformin on differentiation, metformin (dissolved in water) at various concentrations (2.5, 5, and 10 mM) was added to the medium for every 2 days with DMI. After differentiation, 80–90% 3T3-L1 cells showed mature adipocyte phenotype.

### 4.2 RNA-seq and quality control

These libraries were sequenced on the Illumina-HiSeq 2000 platform using a 150 bp paired-end sequencing strategy. The original image data generated by the sequencer is converted into sequence data through base calling (Illumina pipeline CASAVA v1.8.2), and then subjected to standard quality control (QC) standards to remove all reads that meet any of the following parameters: (1) adapters and primer-aligned reads, no more than two mismatches, (2) Unknown bases greater than 5% when reading (3) Low-quality bases greater than 50%. Finally, the filtered readings are reserved for further analysis after quality control.

### 4.3 Differential expression analysis

The DESeq2 R software package was used to perform differential expression analysis on two conditions/groups (three biological replicates for each condition). DESeq2 provided statistical routines for confirming the differential expression in digital gene expression data using models based on the negative binomial distribution. The method of Benjamini and Hochberg was used to adjust the generated P value to control the false discovery rate. Genes found by DESeq2 with a adjusted P value of <0.05 were designated as differentially expressed genes.

### 4.4 GO and KEGG analysis of differentially expressed genes

The cluster Profiler R software package was uesd to perform gene ontology (GO) enrichment analysis of differentially expressed genes. GO with corrected P value is less than 0.05 are considered to be a significant enrichment of differentially expressed genes. KEGG is a database resource used to understand the advanced functions and utility of biological systems, such as cells, organisms, and ecosystems from molecular-level information, especially large-scale molecular data sets generated by genome sequencing and other high-throughput experimental technologies (http://www.genome.jp/kegg/) [30]. We used the cluster Profiler R software package to test the statistical enrichment of differentially expressed genes in the KEGG pathway.

### 4.5 Identification of DEGs of gene expression profile data

The gene expression profile datasets GSE20696, GSE93637, GSE50934, GSE114690, GSE95533, GSE129957 and GSE50612 were downloaded from the GEO database (https://www.ncbi.nlm.nih.gov/geo/) [36–42]. For the seven profile data of adipogensis, HiSat2 and feature counting were used to map the read sequence to the mouse genome (GRCm38), and to quantify the annotated genes [46,47]. The DESeq2 R software package was used for the differential expression analysis. For the original data of GSE93637 and GSE20696, the limma R software package was used for normalization and differential expression analysis. Both DESeq2 and limmar packages are from the Bioconductor project (https://www.bioconductor.org/). All R packages used in this research are deployed in the programming language R (version 3.3.3, Auckland, New Zealand). The bioinformatic analyses of seven expression profile datasets were shown in Table 1.

### 4.6 MTT assay

Cell viability was assessed by MTT (3-(4,5-dimethylthiazol-2-yl)-2,5-diphenyltetrazoliumbromide, Sigma-Aldrich, St. Louis, MO, USA) assay. Cells were seeded in 96-well culture plates at 3 × 10^3^ cells per well for 24 h. Then, different doses of metformin were added into these wells for 48 h. Subsequently, 10 μL of MTT solution (10 mg/mL) was added into each well, and cells were incubated at 37°C for additional 4 h. Next, the supernatant in each well was replaced by dimethyl sulphoxide (DMSO), and the absorbance was measured using microplate reader (Bio-Rad, Hercules, CA) at 550 nm. All the experiments were independently performed at least three times. Cell viability curves were plotted using the absorbance at each time point.

### 4.6 Oil Red O staining

The mature adipocytes were fixed with 4% formaldehyde at 37°C for 10 min, stained with 0.4% Oil Red O in 3:2 (v/v) isopropanol/H2O for 30 minutes at room temperature and then rinsed three times with PBS, observed under inverted light Nikon microscope.

### 4.7 RNA extraction and quantitative PCR

Total RNA was extracted from cells using Trizol reagent (Invitrogen, Carlsbad, CA). RNA was reverse transcribed with PrimeScript RT-PCR Kit (Vazyme Bio, Nanjing, China) to generate cDNA for real-time PCR using SYBR Premix Ex Taq (Vazyme Bio, Nanjing, China) in an ABI Prism 7900HT Sequence Detection System (Thermo Fisher Scientific, USA). The relative expression of mRNA was calculated using the 2-ΔΔCT method. Primers used for real-time PCR are shown in Table 2.

**Table 2.** Sequence of gene primers for fluorescent quantitative PCR

Gene Symbol	Forward primer	Reverse primer
PPARγ	TCGCTGATGCACTGCCTATG	GAGAGGTCCACAGAGCTGATT
C/EBPα	AGACATCAGCGCCTACATCG	TGTAGGTGCATGGTGGTCTG
Scd1	GTCGTACCACTGGCATTGTG	GCCATCTCCTGCTCAAAGTC
FABP4	AAGAAGTGGGAGTGGGCTTT	GCTCTTCACCTTCCTGTCGT
Etfdh	GCCTAGGACATCTGGTGAGC	ACTGTGACTTTGGCATGCAG
Abcg1	GCTGGGAAGTCCACACTCAT	CTCGTCTGCCTTCATCCTTC
Abhd3	TGCCAAGAAACCCCAGTTAG	GAGATCTGTCCTCCGTCTGC
Adhfe1	CCAGCCCTCACTCTGAGTTC	AGAGTGTGCAGGGGATCAAC
Aldh6a1	GATGTATTCCGAGGCCTTCA	TTGCTCCAGGTACTCGCTCT
Aldoa	GAACCAATGGCGAGACAACT	GTCCCCATCAGGGAGAATTT
Bckdhb	AGATGTTGCCTTTGGTGGAG	CTGATCAAAGGCAGGGAAAA
Dbt	CTCCAGTGTTCACAGGCAAA	AACTGCAGGAGTCCCAGAGA
Des	GGATGCAGCCACTCTAGCTC	CTCATACTGAGCCCGGATGT
Egln3	CAGGTTATGTTCGCCATGTG	CATAGGAGGGCTGGACTTCA
Hadhb	CAGCTGTCCAGACCAAGTCA	AACCCGAAAGTGCAGCTCTA
Hibch	GTCCATGGTCAATTCCGAGT	ACACGCAACTTTTCCGAATC
Mrap	CAAGCATTCCATTGTCATCG	CTCTCCTTCCTGGCTCCTCT
Pde3b	AGTACCGCGGAGGAAAAAGT	CTCCATTTCCACCTCCAGAA
Pex13	TCTGGATATGGAGCCTACGG	AGGCAAATGCATGCACAATA
Syn2	GACTTTGTGCTCATCCGACA	TCTCTCGGTGATTGGGGTAG
Tst	CCCACTTTGGGGACTATGTG	TCAGTGTGGCTTTGAAAACG
β-actin	TGGAATCCTGTGGCATCCATGAAAC	TAAAACGCAGCTCAGTAACAGTCCG

### 4.8 Statistical analyses

The experimental data was expressed as the mean ± standard deviation (SD). Statistical differences were evaluated by Student’s t-test for two groups using GraphPad Prism version 5.00 (GraphPad, San Diego, CA, USA), and a p-value <0.05 was considered statistically significant.

## Data Availability

The datasets presented in this study can be found in the Gene Expression Omnibus. The names of the repository is ‘Next Generation Sequencing Facilitates Quantitative Analysis of metformin on adipogenesis Transcriptomes’, the accession number is GSE179531. https://www.ncbi.nlm.nih.gov/geo/query/acc.cgi?acc=GSE179531

## Abbreviations

DMI, Dexamethasone/3-isobutyl-1-methylxanthine/Insulin; GEO, Gene Expression Omnibus; PPARγ, Peroxisome proliferator activated receptor gamma; DEGs, Differentially expressed genes; RRA, Robust rank aggregation; GO, Gene ontology; KEGG, Kyoto encyclopedia of genes and genomes; PCA, Principal component analysis.
